# Whole Exome Sequencing Reveals Novel Variants in Unexplained Erythrocytosis

**DOI:** 10.1089/omi.2023.0059

**Published:** 2023-07-19

**Authors:** Harshit Khurana, Babylakshmi Muthusamy, Uday Yanamandra, Kishore Garapati, Harikrishnan Premdeep, Shankar Subramanian, Akhilesh Pandey

**Affiliations:** ^1^ Command Hospital (Air Force), Bangalore, India.; ^2^ Department of Internal Medicine, Armed Forces Medical College, Pune, India.; ^3^ Institute of Bioinformatics, International Technology Park, Bangalore, India.; ^4^ Department of Laboratory Medicine and Pathology, Mayo Clinic, Rochester, Minnesota, USA.; ^5^ Manipal Academy of Higher Education, Manipal, India.; ^6^ Center for Individualized Medicine, Mayo Clinic, Rochester, Minnesota, USA.

**Keywords:** polycythemia, *JAK2*-negative, mutations, diagnostics, whole exome sequencing, personalized medicine

## Abstract

Erythrocytosis is characterized by an increase in red cells in peripheral blood. Polycythemia vera, the commonest primary erythrocytosis, results from pathogenic variants in *JAK2* in ∼98% of cases. Although some variants have been reported in *JAK2*-negative polycythemia, the causal genetic variants remain unidentified in ∼80% of cases. To discover genetic variants in unexplained erythrocytosis, we performed whole exome sequencing in 27 patients with *JAK2*-negative polycythemia after excluding the presence of any mutations in genes previously associated with erythrocytosis (*EPOR*, *VHL*, *PHD2*, *EPAS1*, *HBA*, and *HBB*). We found that the majority of patients (25/27) had variants in genes involved in epigenetic processes, including *TET2* and *ASXL1* or in genes related to hematopoietic signaling such as *MPL* and *GFIB*. Based on computational analysis, we believe that variants identified in 11 patients in this study could be pathogenic although functional studies will be required for confirmation. To our knowledge, this is the largest study reporting novel variants in individuals with unexplained erythrocytosis. Our results suggest that genes involved in epigenetic processes and hematopoietic signaling pathways are likely associated with unexplained erythrocytosis in individuals lacking *JAK2* mutations. With very few previous studies targeting *JAK2*-negative polycythemia patients to identify underlying variants, this study opens a new avenue in evaluating and managing *JAK2*-negative polycythemia.

## Introduction

Polycythemia is an absolute or relative increase in hemoglobin, hematocrit, or red cell mass levels above defined ranges (Gangat et al., 2021; Wouters et al., 2020). Next-generation sequencing approaches to identify driver mutations have greatly enhanced our ability to identify *JAK2* mutations in polycythemia (polycythemia vera [PV]) (Stuckey and Gómez-Casares, 2021). In these patients, the most common driver mutations, namely V617F in exon 14 and mutations in exon 12, are present in 96% and 3%, respectively. World Health Organization classification criteria for myeloid neoplasms in 2016 have made the diagnosis of PV relatively straightforward (Sabattini et al., 2010).

However, despite extensive evaluation, we frequently encounter a significant subset of *JAK2*-negative patients with no causes of secondary erythrocytosis (Khurana et al., 2020; Mallik et al., 2019). It is imperative to evaluate patients lacking *JAK2* mutations to identify additional genetic causes of erythrocytosis (Jalowiec et al., 2022; Vannucchi, 2017). This is a heterogeneous group of patients with very few insights available in literature. Currently, such patients are often managed in a similar manner as PV, which might not be the most appropriate approach or treatment. Thus, any attempt to determine the exact underlying pathological processes would be helpful for determining more individualized prognostication and clinical management.

Mutations resulting in dysregulation of the oxygen sensing pathway or formation of high oxygen affinity hemoglobin variants can result in polycythemia. The first of such mutations seen in patients with congenital erythrocytosis was reported in 1966 when the patient and the affected family members had an abnormal hemoglobin band on electrophoresis (Charache et al., 1966). Structural analysis revealed it to be an alpha-chain variant. Since then, >100 mutations in the globin genes have been described, which give rise to high-affinity hemoglobin variants.

Most of these mutations are found in the beta-globin gene; they are electrophoretically silent in most cases and are difficult to detect using routine laboratory tests. Mutations involving the oxygen sensing pathway primarily involve three different genes related to this pathway—von Hippel Lindau (*VHL*), hypoxia-inducible factor 2 alpha (*EPAS1*), and prolyl hydroxylase domain protein-2 (*PHD2*) (Lee, 2008). In this study, we sought to evaluate the spectrum of genetic variants in patients with *JAK2*-negative polycythemia using whole exome sequencing.

## Materials and Methods

### Study design

The study is a cross-sectional descriptive study performed in accordance with the Declaration of Helsinki. Written and informed consent was obtained from all participants of this study. This study protocol and consent forms were approved by the institutional ethics committee at the Command Hospital (Air Force) in Bangalore, India, where the patients were seen and recruited.

### Inclusion and exclusion criteria

All newly diagnosed cases of polycythemia, defined as an increased hemoglobin level (>18.5 g/dL in males and 16.5 g/L in females) or an elevated hematocrit (>0.52 in males and 0.48 in females), which has been persistent for at least 2 months were included in the study. Patients with *JAK2*-positive polycythemia, raised erythropoietin (EPO), recent exposure (in past 6 months) to high altitude, chronic pulmonary disease, sleep apnea, right to left cardiac shunt, tumors known to secrete EPO or exogenous administration of EPO or erythropoietin stimulating agents, or those unwilling to sign written informed consent were excluded from the study.

### Patient evaluation

To ascertain the diagnosis of polycythemia, complete blood cell counts were repeated thrice, maximum over 2 months, using an automated counter. Detailed history was taken from all patients and clinical examination performed to rule out acquired causes of polycythemia. All patients were tested for serum EPO levels, molecular testing for the *JAK2* mutations, and bone marrow examinations. Further guided by the clinical background, certain patients were selectively subjected to investigations such as pulmonary function tests, echocardiography, and imaging studies. Hemoglobin cation-exchange high-performance liquid chromatography was done to rule out hemoglobinopathies or high-affinity Hb. A total of 27 diagnosed cases of *JAK2-*negative polycythemia were recruited to the study.

### Exome sequencing and data analysis

Blood samples were collected from the affected individuals after signing appropriate informed consent forms. Genomic DNA was extracted using standard procedures. Exome sequencing libraries were prepared using SureSelectXT Human All Exon V5+UTR kit (Agilent Technologies, Santa Clara, CA, USA). The captured libraries were sequenced using Illumina HiSeq2000 (Illumina, Inc., San Diego, CA, USA), and paired-end reads (2 × 150 bp) were generated targeting 100 × depth of coverage. Sequencing data analysis and annotation were carried out as described previously (Muthusamy et al., 2017).

Variants were filtered based on the following criteria: a minimum read depth of 20, a minimum base quality score of 20, a minimum mapping quality of 40 and a strand bias threshold of 0.05. These filtering thresholds were applied sequentially to select reliable variants and reduce false positives. The annotated single nucleotide variants and small short insertions/deletions (indels) were further filtered to identify potentially pathogenic variants. After quality filtering of the variants, those located in the exonic and splice site regions were retained.

Common variants with a minor allele frequency of >0.01 were removed after comparing with variants in the 1000 Genomes Project and gnomAD (Gudmundsson et al., 2021). The filtered variants were manually curated, looking for potentially pathogenic variants causing polycythemia. The pathogenicity of the variants was assessed based on protein functions, domains, and conservation of variant location across the species. Any association of variants in the Catalogue of Somatic Mutations in Cancer (COSMIC) and Online Mendelian Inheritance in Man (OMIM) was noted (Amberger et al., 2015; Tate et al., 2019). Furthermore, functional effects of the variants were annotated using Sorting Intolerant from Tolerant and Polyphen-2 and literature curation (Adzhubei et al., 2010; Kumar et al., 2009). Furthermore, the shortlisted variants were assessed for accuracy by visualizing them on the Integrative Genomics Viewer.

### Statistical analysis

Descriptive statistics (measures of central tendency, distribution, and variability) were used to summarize data. Continuous variables were expressed as mean ± standard deviation (SD) with range and categorical variables as proportions.

## Results and Discussion

The patients were screened for *JAK2*-V617F and *JAK2*-exon12 variants using polymerase chain reaction amplification of the target region followed by Sanger sequencing. Out of 28 patients identified as negative for *JAK2*-V617F and all known *JAK2*-exon 12 mutations after initial screening, a repeat evaluation revealed exon 12 deletion in one patient who was then excluded from whole exome sequencing. Other genes that were similarly tested for known mutations include *EPOR*, *VHL*, *PHD2*, *EPAS1*, *HBA*, and *HBB*, and all study subjects were found to be negative for them. A total of 27 patients were finally subjected to whole exome sequencing (WES).

The median age of study participants was 34 years (21–84) with a male preponderance (96.3%) as they were all military personnel. The baseline characteristics of the study participants are shown in Table 1. The mean hemoglobin and packed cell volume were 19.0 ± 0.9 g/dL and 55.2 ± 4.01%, respectively. Total leukocyte and platelet counts were within normal range in all patients with a mean (SD) of 7345 ± 1746/μL and 260,107 ± 64,167/μL, respectively. Two study participants had pulmonary pathology; however, none had hypoxemia (normal SPO_2_ and arterial blood gas analysis in all patients except in one patient who had alkalosis of uncertain significance). Echocardiography did not reveal right to left shunts in any of the patients.

**Table 1.** Baseline Characteristics of Patients Included in This Study

Characteristic	All patients* n* = 27
Smoking, number (%)	
Current smoker	1 (3.7)
Former smoker	6 (22.2)
Never smoked	20 (74.1)
Family history of polycythemia, number (%)	
Present	1 (3.7)
Absent	26 (96.3)
Place of residence, number (%)	
Plain—not high altitude	25 (92.6)
High altitude area but resided on the plains (not high altitude) in the prior 6 months	2 (7.4)
Exposure to high altitude area within the prior 6 months	None
Hemoglobin (g/dL)	
Mean (SD)	19.01 (0.91)
Packed cell volume	
Mean (SD)	55.2 (4.01)
Total leucocyte count ( × 10^3^/μL)	
Mean (SD)	7.3 (1.7)
Platelets ( × 10^3^/μL)	
Mean (SD)	260 (65)
Bone marrow studies, number (%)	
Normal	08 (29.6%)
Erythroid hyperplasia	19 (70.4%)
Lung disease, number (%)	
Not present	26 (96.3)
Present	1 (3.7%)
Cardiovascular ailments, number (%)	
Not present	21 (77.7)
Present	6 (22.2)

Among the 27 study participants who underwent WES, 24 patients were found to have variants affecting several genes involved in epigenetic processes and hematopoietic signaling pathways. A total of 84 high confidence variants (with minimum depth of coverage of 20 × ) were identified, out of which 66 (79%) were classified as probably nonpathogenic, 11 (13%) as possibly pathogenic, and 7 (8%) as probably pathogenic based on prediction tools.

**FIG. 1.**
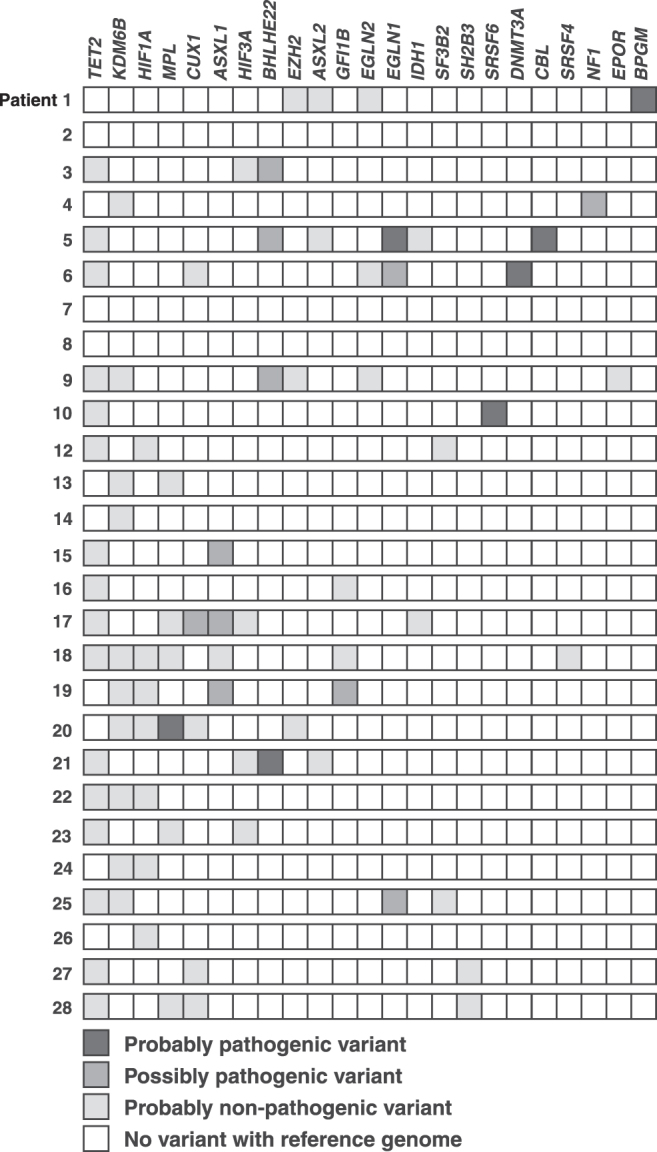
Genes involved in epigenetic processes and oxygen sensing pathways, in which variants were identified in patients in this study. Each *row* represents a patient with the columns indicating genes as shown. The *boxes* are *shaded* as indicated in the figure to reflect the predicted effect of the identified variants. *ASXL1*, additional sex combs like 1; *ASXL2*, additional sex combs like 2; *BHLHE22*, basic helix-loop-helix family member e22; *BPGM*, bisphosphoglycerate mutase; *CBL*, casitas B-lineage lymphoma; *CUX1*, cut like homeobox 1; *DNMT3A*, DNA methyltransferase 3 alpha; *EGLN1*, egl-9 family hypoxia inducible factor 1; *EGLN2*, egl-9 family hypoxia inducible factor 2; *EPOR*, erythropoietin receptor; EZH2, enhancer of zeste homolog 2; *GFI1B*, growth factor independent 1B transcriptional repressor; *HIF1A*, hypoxia inducible factor subunit 1 alpha; *HIF3A*, hypoxia inducible factor subunit 3 alpha; *IDH1*, isocitrate dehydrogenase 1; *KDM6B*, lysine(K) demethylase 6B; *MPL*, myeloproliferative leukemia virus; *NF1*, neurofibromatosis type 1; *SF3B2*, splicing factor 3B subunit 2; *SH2B3*, SH2B adaptor protein 3; *SRSF4*, serine and arginine rich splicing factor 4; *SRSF6*, serine and arginine rich splicing factor 6; *TET2*, ten–eleven translocation-2.

Genes that were identified with probably pathogenic variants in these 24 patients included *EGLN1*, *CBL*, *DNMT3A*, *MPL*, *BPGM*, *BHLHE22*, and *SRSF6*; those with possibly pathogenic variants included *CUX1*, *ASXL1*, *BHLHE22*, *EGLN1*, *GFI1B*, and *NF1*. Notably, possibly pathogenic variants affecting *ASXL1* were found in three patients, whereas two patients had possibly pathogenic variants in *EGLN1*. Variants predicted to be probably nonpathogenic variants were most often observed in the *TET2* gene (*n* = 16 patients). Nonpathogenic variants were also identified involving *KDM6B* (*n* = 10) and *HIF-1α* (*n* = 7) (Fig. 1).

With very few previous studies targeting *JAK2*-negative polycythemia patients to identify underlying variants, our study opens a new avenue in evaluating and managing *JAK2*-negative polycythemia. This study revealed variants in genes involved in epigenetic processes and hematopoietic signaling pathways with a potential role in the pathogenesis of *JAK2*-negative polycythemia. We discuss herein some of the identified variants characterized as probably pathogenic and their predicted downstream molecular consequences.

**FIG. 2.**
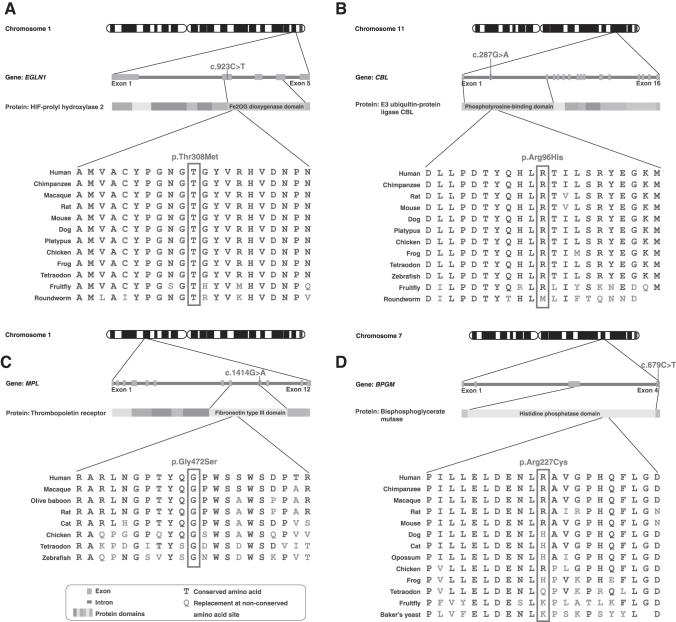
Genetic variants and their predicted deleterious changes in the encoded proteins. **(A)** Variant c.923C>T in the coding region of *EGLN1* (patient 5) leads to a predicted protein-level change p.Thr308Met, which is in the catalytic dioxygenase domain of the encoded protein**. (B)** Variant c.287G>A in the coding region of *CBL* (patient 5) leads to a predicted protein-level change of p.Arg96His in a highly conserved amino acid in its phosphotyrosine-binding domain of Cbl. **(C)** Variant c.1414G>A in the coding region of *MPL* (patient 20), with a predicted protein-level change in a highly conserved amino acid in thrombopoietin receptor. **(D)** Variant c.679C>T in *BPGM* (patient 1), which leads to a predicted protein-level change in a highly conserved amino acid in the histidine phosphatase domain of bisphosphoglycerate mutase.

Among the probably pathogenic variants, *EGLN1* c.923C>T; p.Thr308Met, identified in patient 5, is in the catalytic domain of the protein with the amino acid Thr308 being highly conserved across species (Fig. 2A). This variant is predicted to be disease-causing and might affect proline hydroxylation of hypoxia inducible factor (HIF)-1α. The protein encoded by the *EGLN1* gene is involved in the hydroxylation of proline in HIF-α, which is critical for oxygen homeostasis. As a cellular oxygen sensor, under normoxic conditions, it regulates the prolyl hydroxylation of HIF subunits through the VHL complex, and mutations affecting this gene are implicated in erythrocytosis familial type 3 (ECYT3) (Hirota, 2021; Lee, 2008). In previous studies, variants in genes encoding the VHL complex have been associated with congenital polycythemia (Ang et al., 2002a; Ang et al., 2002b).

The same patient with the variant in *EGLN1* (patient 5) also possessed a probably pathogenic variant in *CBL*, (c.287G>A; p.Arg96His) which is a proto-oncogene encoding a RING finger E3 ubiquitin ligase. The N-terminal of Cbl contains a phosphotyrosine binding domain, which facilitates proteasomal degradation of tyrosine-phosphorylated substrates (Fig. 2B). Previous studies have implicated its role in the causation of myeloproliferative neoplasms (MPN), including *JAK2*-positive polycythemia (Aranaz et al., 2012). The variant identified in this patient involves a change in a highly conserved amino acid within the N-terminal adaptor helical domain, which is predicted to be disease-causing. This variant in *CBL* is listed in the COSMIC database in the causation of adenocarcinoma colon with no record of polycythemia (Forbes et al., 2017).

Sequencing of patient 25, reportedly of semi-high-altitude residence, identified a variant in *EGLN1*, c.12C>G; p.Asp4Glu. The amino acid Asp4 is weakly conserved across species and this change is predicted to be benign. Interestingly, in cases where this variant occurs along with another variant in the same gene (c.380G>C; p.Cys127Ser, which was not identified in this patient), it is associated with high altitude adaptation hemoglobin and is termed the Tibetan haplotype (Lorenzo et al., 2014). We also identified a nonsense variant (c.958C>T; p.Arg320*) in the *DNMT3A* gene in patient 6, which is predicted to lead to premature termination of the protein product at Arg320 that may result in nonsense-mediated decay and loss of gene function. *DNMT3A* codes for mammalian DNA methyl transferase, an enzyme involved in epigenetic processes.

In previous studies, variants in *DNMT3A* have been associated with MPN, including *JAK2*-positive polycythemia (Easwar and Siddon, 2021; Kjaer, 2020; Loscocco et al., 2021). We identified a missense variant in *MPL* in patient 20 (c.1414G>A; p.Gly472Ser). *MPL* is a proto-oncogene known to play a crucial role in thrombopoiesis through the Janus kinase/signal transducers and activators of transcription (JAK-STAT) signaling pathway (Fig. 2C). This variant is predicted to be disease-causing with variants in this gene previously associated with MPN.

In patient 1, we identified a variant in a conserved amino acid in *BPGM* (c.679C>T; p.Arg227Cys) predicted to be disease-causing (Fig. 2D). *BPGM* codes for bisphosphoglycerate mutase, an enzyme that decreases the oxygen affinity of hemoglobin. Previous studies have reported several variants in *BPGM* as a cause of erythrocytosis.

Our study also identified probably and possibly pathological variants involving the *BHLHE22* gene. This gene codes for a protein essential in cell proliferation and differentiation. The role of this gene in the pathogenesis of polycythemia has not been reported previously. We also found a probably pathogenic variant in *SRSF6* in patient 10. This gene is involved in mRNA splicing, and although none of the variants involving this gene have been related to polycythemia in previous studies, cases have been reported where splicing defects were associated with *MPN* (Hautin et al., 2020; Makishima et al., 2012). Apart from the probably pathogenic variants, many possibly pathogenic variants were also identified in our study. Establishing the roles of these variants in the pathogenesis of MPN requires further experimental and bioinformatic analysis.

This study adequately ruled out all possible secondary causes of polycythemia. Smoking was ruled out as a cause of polycythemia owing to low EPO levels in all but one patient, who was a current smoker at the time of examination. For this patient, follow-up after cessation of smoking will be necessary as no pathogenic variants were identified. The lack of serial hemoglobin values before and after inclusion in the study is a potential limitation.

To our knowledge, this is the largest study of genetic variants in individuals with unexplained erythrocytosis. Our results strongly suggest that in patients with *JAK2*-negative polycythemia with no identifiable secondary causes of erythrocytosis, there might be novel genetic variants that could be implicated in its pathogenesis. Further studies could provide novel diagnostic and therapeutic opportunities for better management of such cases.

## Abbreviations Used

COSMIC: Catalogue of Somatic Mutations in Cancer
EPO: erythropoietin
HIF: hypoxia inducible factor
MPN: myeloproliferative neoplasms
PV: polycythemia vera
SD: standard deviation
VHL: von Hippel Lindau
WES: whole exome sequencing
